# Yogurt consumption and risk of accelerated aging: A population-based study from the NHANES 2003–2006

**DOI:** 10.3389/fnut.2024.1482980

**Published:** 2024-12-11

**Authors:** Xinghai Yue, Hongfei Liu, Wenzhao Guo, Yuhang Gao, Shaoshun Shi

**Affiliations:** ^1^The Second Clinical College, Liaoning University of Traditional Chinese Medicine, Shenyang, China; ^2^Department of Intensive Care Unit, The Second Affiliated Hospital of Liaoning University of Traditional Chinese Medicine, Shenyang, China

**Keywords:** yogurt, aging, phenotypic age, body mass index, overweight status

## Abstract

**Introduction:**

Yogurt consumption is beneficial to health, but its association with aging remains unclear. This study aims to explore the relationship between yogurt consumption and aging using data from the 2003–2006 National Health and Nutrition Examination Survey (NHANES).

**Methods:**

We used data from 4,056 participants to examine the relationship between yogurt consumption and aging. Yogurt consumers were defined as individuals who consumed yogurt at least once per year. Phenotypic age acceleration was used as a surrogate marker for aging. Nearest-neighbor propensity score matching (PSM) was applied to reduce bias, followed by weighted multivariate logistic regression analysis, subgroup analysis, and restricted cubic spline (RCS) to investigate the relationship between yogurt consumption and aging.

**Results:**

Yogurt consumption was associated with a lower risk of accelerated aging compared to non-consumers (OR = 0.544, 95% CI: 0.354–0.836, *p* = 0.020). A U-shaped relationship was observed between the frequency of yogurt intake and the risk of accelerated aging. Additionally, yogurt consumption was related to a lower risk of overweight status.

**Discussion:**

These findings suggest that yogurt consumption may reduce the risk of accelerated aging and may also be linked to a lower risk of overweight status. This could provide a promising avenue for exploring the beneficial effects of dietary factors on lifespan extension.

## 1 Introduction

Aging is one of the risk factors for diabetes, coronary heart disease, cancer, and various neurological diseases. With the global progression of aging, the associated disease burden has become increasingly significant, making aging an important health issue that the medical community cannot ignore (1, 2). Therefore, finding ways to delay aging and reduce the resulting disease burden, thereby improving the quality of life and lifespan of the elderly, is a crucial challenge that the healthcare field must address.

Dietary therapy is a popular research topic in the field of aging (3, 4). This approach is more convenient, safer, and low-cost (3, 5). Researchers have long been committed to achieving the goal of preventing or treating diseases and delaying aging through dietary interventions. Fermented dairy products play an important role in this regard (6), and many studies have demonstrated the beneficial health effects of fermented dairy consumption. In a large-scale cohort study conducted in the Netherlands in 2011 (*n* = 120,825), a negative correlation between fermented dairy product intake and all-cause mortality was demonstrated among participants (7). Similarly, a 12-year cohort study by Sonestedt et al. (8) found a significant inverse association between fermented dairy intake and the incidence of cardiovascular disease. The group with the highest intake of fermented dairy had a 15% lower risk of cardiovascular disease compared to the group with the lowest intake (95% CI: 5–24%; *p* trend = 0.003).

As a type of fermented dairy product, yogurt is rich in nutrients and probiotics. Compared to milk, it contains higher levels of protein, vitamin B2, vitamin B12, calcium, magnesium, potassium, and zinc (9). It is also a component of the Mediterranean diet (10). Moreover, the probiotics in yogurt can promote lactose absorption and alleviate the symptoms of lactose intolerance (11), indicating that yogurt is more suitable for a wider population than milk. Yogurt has been shown in numerous studies to have various positive effects on human health (12). For example, studies have shown a negative correlation between yogurt consumption and the prevalence of hypertension (13) and diabetes (14). Two studies indicated that, among the elderly, yogurt consumers had better cognitive function compared to non-consumers, including better memory, executive function, and verbal fluency (15, 16). And a 2017 study from Ireland (*n* = 4,310) supported the association between yogurt consumption and bone health (17). This study demonstrated that higher yogurt intake was associated with a lower risk of osteoporosis and fractures, as well as better Timed Up and Go (TUG) performance. Subsequently, a 2021 study from Canada (*n* = 7,945) further confirmed these findings (18).

Phenotypic age acceleration was first proposed by Buendia et al. (13), and this metric is derived from nine commonly used clinical biomarkers along with chronological age. Phenotypic age is a measure that reflects an individual’s estimated mortality risk, corresponding to their expected age within the population. This metric is widely used in the literature as an indicator for identifying morbidity and mortality risk factors, evaluating intervention outcomes, and elucidating mechanisms of aging. Therefore, in this study, we used phenotypic age acceleration as a surrogate marker for aging in our analysis.

NHANES is a cross-sectional database conducted by U.S. agencies, collecting comprehensive health, nutrition, and lifestyle data. The study has been ongoing since 1999, and it is widely used for research in fields like public health and epidemiology due to its rich, representative dataset (19).

In summary, numerous studies have demonstrated a close association between yogurt and various diseases, as well as physical functions in the elderly, but whether yogurt itself is linked to aging remains unclear. Therefore, this study aims to explore the specific association between yogurt consumption and aging using data from the 2003–2006 NHANES, with the goal of providing clinical insights.

## 2 Methods

### 2.1 Study population

The individual data used in this study were sourced from the 2003–2006 NHANES database, including individuals who had complete dietary frequency questionnaire results, body mass index (BMI), and the biomarkers required for calculating phenotypic age (20). All participants in this study provided informed consent, and the study was approved by the Ethics Review Board of the National Center for Health Statistics.^1^ The specific NHANES codes for the data used in this study can be found in the Supplementary Table S1.

### 2.2 Definition of yogurt consumer

Data on yogurt consumption were obtained from the dietary frequency questionnaire, with the variable identifier FFQ0108: “How often did you eat yogurt (NOT including frozen yogurt)?” This variable contains 11 valid values, each representing a different frequency, where 1 corresponds to “never” and 11 corresponds to “two or more times per day.” We defined yogurt consumers as individuals with values ranging from 2 to 11 (i.e., those who consumed yogurt at least once per year).

[Yogurt consumption frequency meanings: (1) never, (2) 1–6 times per year, (3) 7–11 times per year, (4) 1 time per month, (5) 2–3 times per month, (6) 1 time per week, (7) 2 times per week, (8) 3–4 times per week, (9) 5–6 times per week, (10) 1 time per day, (11) 2 or more times per day].

### 2.3 Phenotypic accelerated aging

The nine biomarkers required to calculate phenotypic age acceleration include albumin, creatinine, glucose, C-reactive protein, lymphocyte percent, mean cell volume, red blood cell distribution width, alkaline phosphatase, and white blood cell count. The calculation of phenotypic age acceleration is based on the residuals of phenotypic age after adjusting for chronological age through linear regression. Participants with phenotypic age acceleration greater than 0 were defined as experiencing accelerated aging, while those with phenotypic age acceleration less than 0 were defined as experiencing decelerated aging. The detailed calculation method can be found in the correction associated with the paper (21).

### 2.4 Covariates

In this study, we also selected the following covariates based on references to other literature related to aging. These include sex, race, education level, marital status, poverty income ratio (PIR), BMI, serum cotinine levels, alcohol consumption status, self-reported hypertension, self-reported coronary heart disease, self-reported diabetes, self-reported cancer, low-density lipoprotein (LDL), and total cholesterol (TCHOL) levels. The PIR was used as a proxy for economic status, and serum cotinine levels were used as a proxy for smoking status (22, 23). For marital status, we categorized “Married” and “Living with partner” as “Non-single,” and “Widowed,” “Divorced,” “Separated,” and “Never married” as “Single.”

### 2.5 Statistical analysis

The weighted analysis in this study was conducted using the special weights derived from the dietary frequency questionnaire—WTS_FFQ—and these weights were appropriately calculated based on the survey cycles. To minimize errors arising from uneven group distribution in cross-sectional data, all participants were divided into two groups based on yogurt consumption—consumers and non-consumers. After propensity score matching, analyses were conducted, including multivariable logistic regression for the association between yogurt consumption and phenotypic age acceleration, subgroup analysis, RCS analysis, and analysis of the association between yogurt consumption and overweight status. All analyses in this study were performed under weighted conditions.

The R packages used in this paper include survey (4.4.2), gtsummary (2.0.2), and MatchIt (4.4.5). After completing the statistical analysis, forestploter (1.1.2) was used to create forest plots. The statistical analyses in this study were conducted using R version 4.4.1.

## 3 Results

### 3.1 The characteristics of participants before and after PSM

The characteristics before and after matching are shown in Table 1. A total of 4,056 participants were included in this study, with 2,635 yogurt consumers (65%) and 1,421 non-consumers (35%). Before matching, the average age of the yogurt consumption group was 42.00 ± 18.62 years, with a higher proportion of females (60%) and 40% males. The average BMI was 27.64 ± 6.76, and 21% of the group exhibited phenotypic age acceleration. In contrast, the non-yogurt consumption group had an average age of 46.29 ± 20.09 years, with a higher proportion of males (61%) and 39% females. The average BMI was 28.38 ± 7.61, and 30% of the group exhibited phenotypic age acceleration. The results regarding gender and socioeconomic status (PIR index) were consistent with previous studies, showing that yogurt consumers tend to have a higher proportion of females and individuals with higher socioeconomic status (24, 25).

**Table 1:** Characteristics of full and propensity score-matched cohorts by yogurt consumer.

	Before matching			After matching		
Variables	Yogurt consumer	Non-yogurt consumer	*p*	Yogurt consumer	Non-yogurt consumer	*p*
*n*	2,635	1,421		1,287	1,287	
Age [mean (SD)]	42.00 (18.62)	46.29 (20.09)	<0.001	44.60 (18.77)	45.56 (20.31)	0.347
LDL [mean (SD)]	112.62 (34.90)	111.52 (35.66)	0.581	112.72 (34.49)	111.20 (35.33)	0.456
TCHOL [mean (SD)]	194.38 (41.67)	193.68 (41.54)	0.757	194.28 (41.46)	193.63 (41.66)	0.780
PIR [mean (SD)]	3.13 (1.59)	2.74 (1.55)	0.046	3.06 (1.58)	2.76 (1.55)	0.006
BMI [mean (SD)]	27.64 (6.76)	28.38 (7.61)	0.018	27.94 (6.60)	28.36 (7.68)	0.236
Log2 cotinine [mean (SD)]	−1.86 (5.11)	0.44 (5.85)	<0.001	−0.14 (5.71)	−0.08 (5.72)	0.858
Phenotypic accelerated aging (%)			<0.001			0.001
Yes	556 (21)	433 (30)		301 (23)	370 (29)	
No	2,079 (79)	988 (70)		986 (77)	917 (71)	
Sex (%)			<0.001			0.243
Female	1,578 (60)	560 (39)		588 (43)	548 (43)	
Male	1,057 (40)	861 (61)		699 (57)	739 (57)	
Race (%)			<0.001			0.976
Mexican American	652 (25)	246 (17)		254 (20)	239 (19)	
Non-Hispanic Black	532 (20)	450 (32)		377 (29)	363 (28)	
Non-Hispanic White	1,251 (47)	644 (45)		580 (45)	604 (47)	
Other Hispanic	75 (3)	29 (2)		26 (2)	29 (2)	
Other Race	125 (5)	52 (4)		50 (4)	52 (4)	
Education (%)			<0.001			<0.001
Above high school	1,026 (39)	366 (26)		503 (53)	335 (38)	
High school or equivalent	394 (15)	312 (22)		233 (25)	265 (30)	
Under high school	361 (14)	335 (24)		207 (22)	287 (32)	
Marital status (%)			0.649			0.406
Non-single	1,210 (51)	649 (49)		622 (53)	569 (47)	
Single	1,180 (49)	689 (51)		562 (47)	635 (53)	
Alcohol drinker (%)			0.002			0.094
Yes	1,118 (76)	558 (67)		593 (74)	477 (67)	
No	354 (24)	275 (33)		210 (26)	239 (33)	
Coronary heart disease (%)			0.048			0.331
Yes	70 (4)	62 (6)		48 (5)	54 (6)	
No	1,703 (96)	949 (94)		889 (95)	831 (94)	
Cancer (%)			0.792			0.524
Yes	169 (9)	115 (11)		99 (11)	101 (11)	
No	1,610 (91)	900 (89)		843 (89)	788 (89)	
Hypertension (%)			<0.001			0.120
Yes	596 (27)	438 (36)		362 (33)	374 (34)	
No	1,587 (73)	785 (64)		744 (67)	716 (66)	
Diabetes (%)			0.769			0.043
Yes	183 (7)	123 (9)		115 (9)	103 (8)	
No	2,450 (93)	1,296 (91)		1,172 (91)	1,182 (92)	

SD, standard deviation; PIR, poverty impact ratio; BMI, body mass index; LDL, low-density lipoprotein; TCHOL, total cholesterol.

After matching, the average age of the yogurt consumption group was 44.60 ± 18.77 years, with 57% males and 43% females. The average BMI was 27.94 ± 6.60, and 23% of the group exhibited phenotypic age acceleration. In the matched non-yogurt consumption group, the average age was 45.56 ± 20.31 years, with 57% males and 43% females. The average BMI was 28.36 ± 7.68, and 29% of the group exhibited phenotypic age acceleration.

Before matching, there were significant differences between the two groups in terms of age, PIR, BMI, serum cotinine levels, sex, race, education, alcohol consumption, coronary heart disease, and hypertension. After PSM, the two groups only differed in PIR, education, and diabetes.

It is important to note that we used PSM to eliminate potential bias due to groupings in the cross-sectional data. However, one limitation of this study is that, even after PSM, significant differences remained between the two groups in terms of PIR index and education level. Given that individuals with better economic conditions and higher education levels may have healthier dietary conditions and habits, caution is needed when interpreting our study’s conclusions.

### 3.2 Analysis of risk factors for accelerated aging

**Figure 1:**
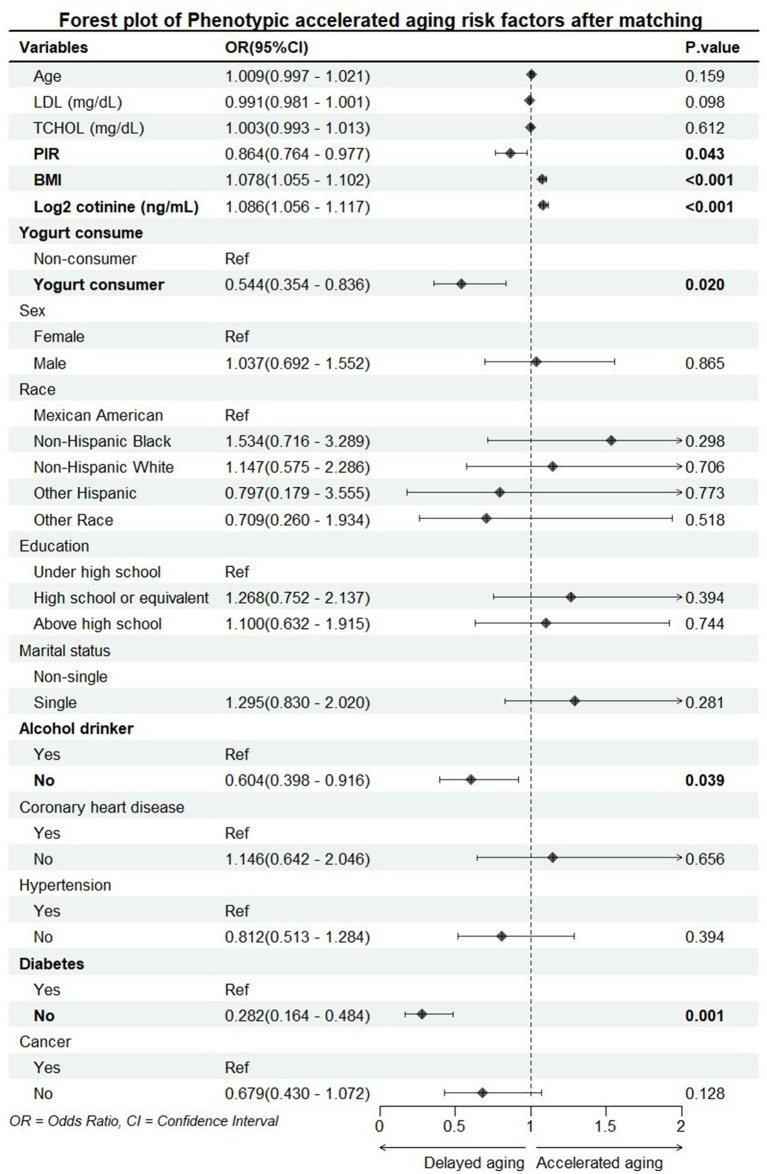
Forest plot of accelerated aging risk factors. PIR, poverty impact ratio; BMI, body mass index; LDL, low-density lipoprotein; TCHOL, total cholesterol.

According to the results of the multivariate logistic regression analysis shown in Figure 1, yogurt consumers had a lower risk of accelerated aging compared to non-consumers [OR (95%) = 0.544 (0.354–0.836), *p* = 0.020]. This result is consistent with the conclusions mentioned in the introduction, indicating that yogurt consumption may have a protective effect on human health.

PIR, BMI, serum cotinine levels, alcohol consumption, and diabetes were all significantly associated with accelerated aging. PIR is used as a proxy for socioeconomic status, serum cotinine levels as a proxy for smoking, and alcohol consumption—these three variables have been confirmed in multiple studies to be associated with aging. Additionally, the close relationship between BMI and accelerated aging has also been validated by numerous studies (26, 27). Furthermore, since one of the nine biomarkers required for calculating phenotypic age acceleration is blood glucose, and the diabetes data in this study are based on self-reported information, there may be bias in the results regarding the association between diabetes and accelerated aging.

### 3.3 Subgroup analysis

**Figure 2:**
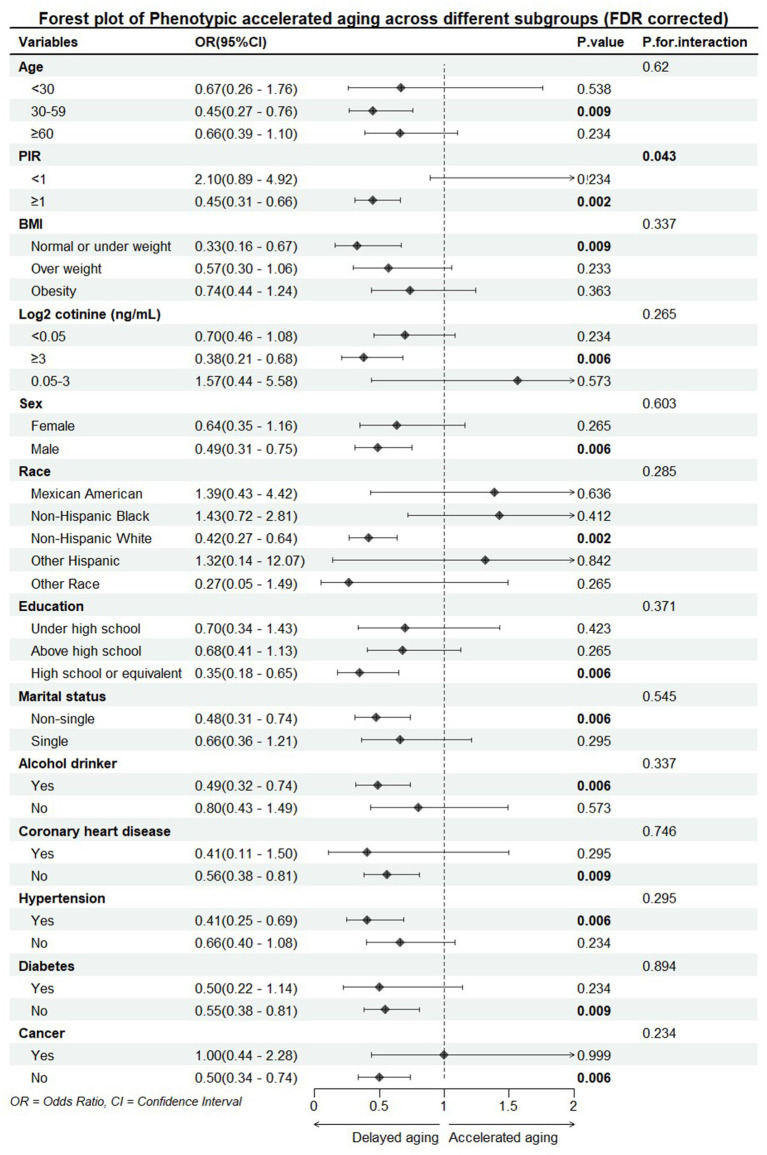
Forest plot of subgroup analysis of the association between yogurt consumption and phenotypic accelerated aging. PIR, poverty impact ratio; BMI, body mass index. All models were adjusted for the following covariates: sex, age (<30, 30–59, ≥60), log2 cotinine (<0.05, 0.05–3, ≥3), LDL, TCHOL, PIR (<1, ≥1), BMI (normal or underweight, overweight, obesity), race/ethnicity (Mexican American, Non-Hispanic White, Non-Hispanic Black, Other Hispanic, Other race), education level (less than high school, high school or equivalent, more than high school), marital status (non-single, single), alcohol consumption (yes, no), diabetes (yes, no), high blood pressure (yes, no), coronary heart disease (yes, no), and cancer (yes, no).

Subsequently, we investigated the association between yogurt consumption and accelerated aging across different subgroups. We categorized age into three groups: “<30,” “30–59,” and “≥60,” and divided PIR into two groups: “<1” and “≥1.” BMI was categorized into “normal or underweight (<25),” “overweight (25–30),” and “obese (≥30).” Log2 cotinine was divided into three groups: “<0.05,” “0.05–3,” and “≥3” (15). And the previous groupings, including sex, race/ethnicity (Mexican American, Non-Hispanic White, Non-Hispanic Black, Other Hispanic, Other race), education level (less than high school, high school or equivalent, more than high school), marital status (non-single, single), alcohol consumption (yes, no), diabetes (yes, no), high blood pressure (yes, no), coronary heart disease (yes, no), and cancer (yes, no). We also explored the interaction between these subgroups and yogurt consumption. All *p*-values were adjusted using false discovery rate (FDR) correction to ensure reliability, and the results are shown in Figure 2.

The results of the subgroup analysis indicate that the association between yogurt consumption frequency and accelerated aging varies significantly across different groups. A significant association was observed only in individuals aged 30–59, males, Non-Hispanic Whites, those with a PIR index ≥1, those who are underweight or of normal weight, individuals with log2 cotinine levels ≥3 ng/mL, those with a high school or equivalent education, single individuals, alcohol drinkers, those with hypertension, non-cardiovascular disease patients, non-diabetic individuals, and non-cancer individuals. We also found a significant interaction between PIR index and yogurt consumption. We speculate that this association may be due to the relatively better access to healthcare and nutritional resources in the group with PIR ≥1.

### 3.4 Nonlinear association

**Figure 3:**
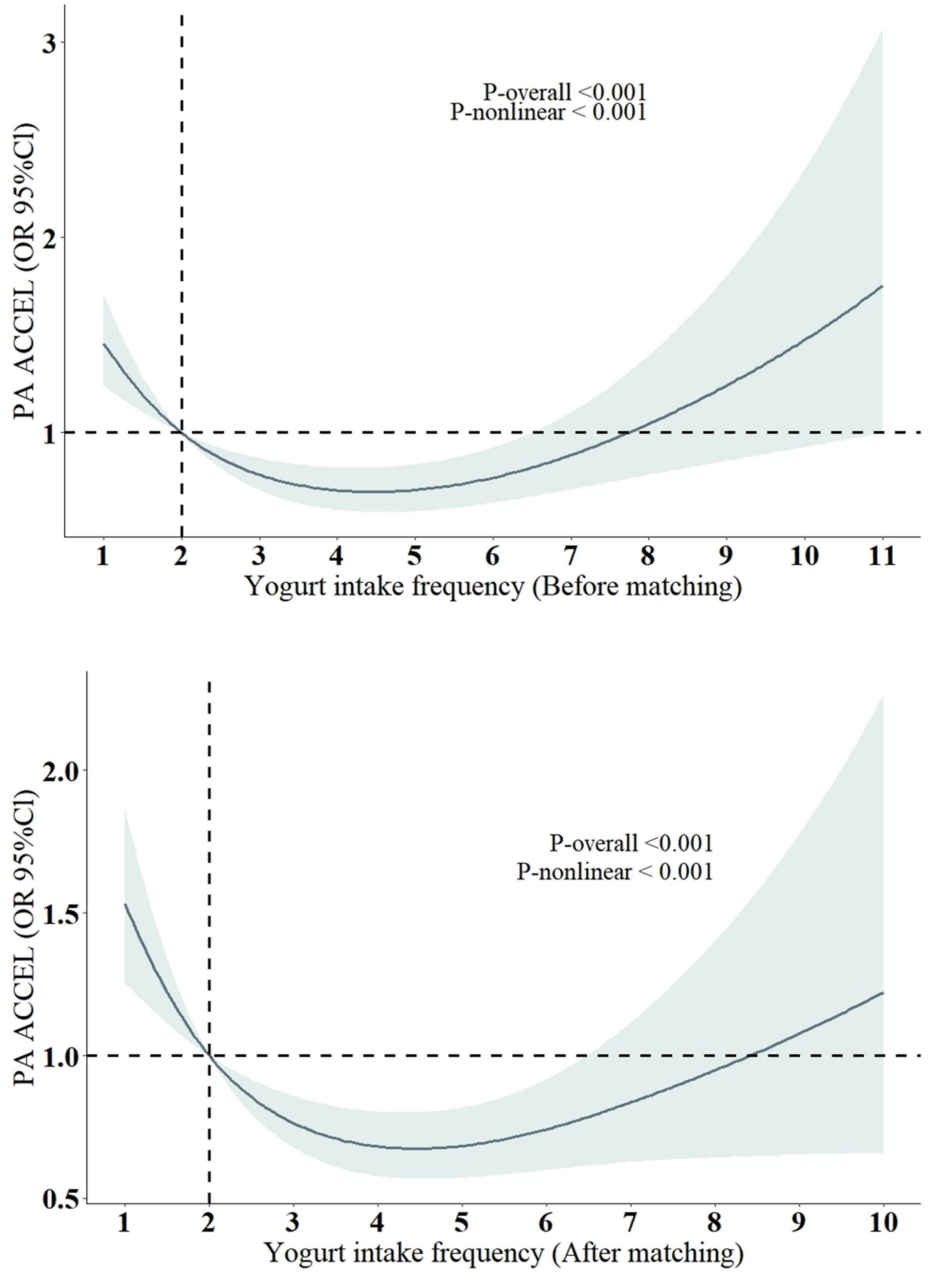
Restricted cubic spline models for the relationship between yogurt intake frequency and the risk of Phenotypic accelerated aging. All models were adjusted for the following covariates: sex, age, BMI, log2 cotinine, LDL, TCHOL, PIR, race/ethnicity (Mexican American, Non-Hispanic White, Non-Hispanic Black, Other Hispanic, Other race), education level (less than high school, high school or equivalent, more than high school), marital status (non-single, single), alcohol consumption (yes, no), diabetes (yes, no), high blood pressure (yes, no), coronary heart disease (yes, no), and cancer (yes, no).

We used RCS to explore the potential nonlinear association between yogurt consumption frequency and accelerated aging and conducted analyses both before and after PSM. The results from both analyses were consistent (Figure 3), indicating a U-shaped relationship between yogurt consumption frequency and the risk of accelerated aging. This suggests that an appropriate frequency of yogurt consumption may help delay aging.

However, due to the lack of specific yogurt intake data in our study, the relevance of consumption frequency alone, without considering the amount consumed, is limited and requires further verification. A recent study (24) indicated a similar J-shaped association between yogurt consumption and hepatic steatosis (HS) and provided a recommended intake of 0.4 cups per day, which is approximately 95 mL/day (1 cup = 237 mL). But this study was also based on cross-sectional NHANES data, so the results should be interpreted with caution.

### 3.5 Yogurt consumption with overweight status

**Figure 4:**
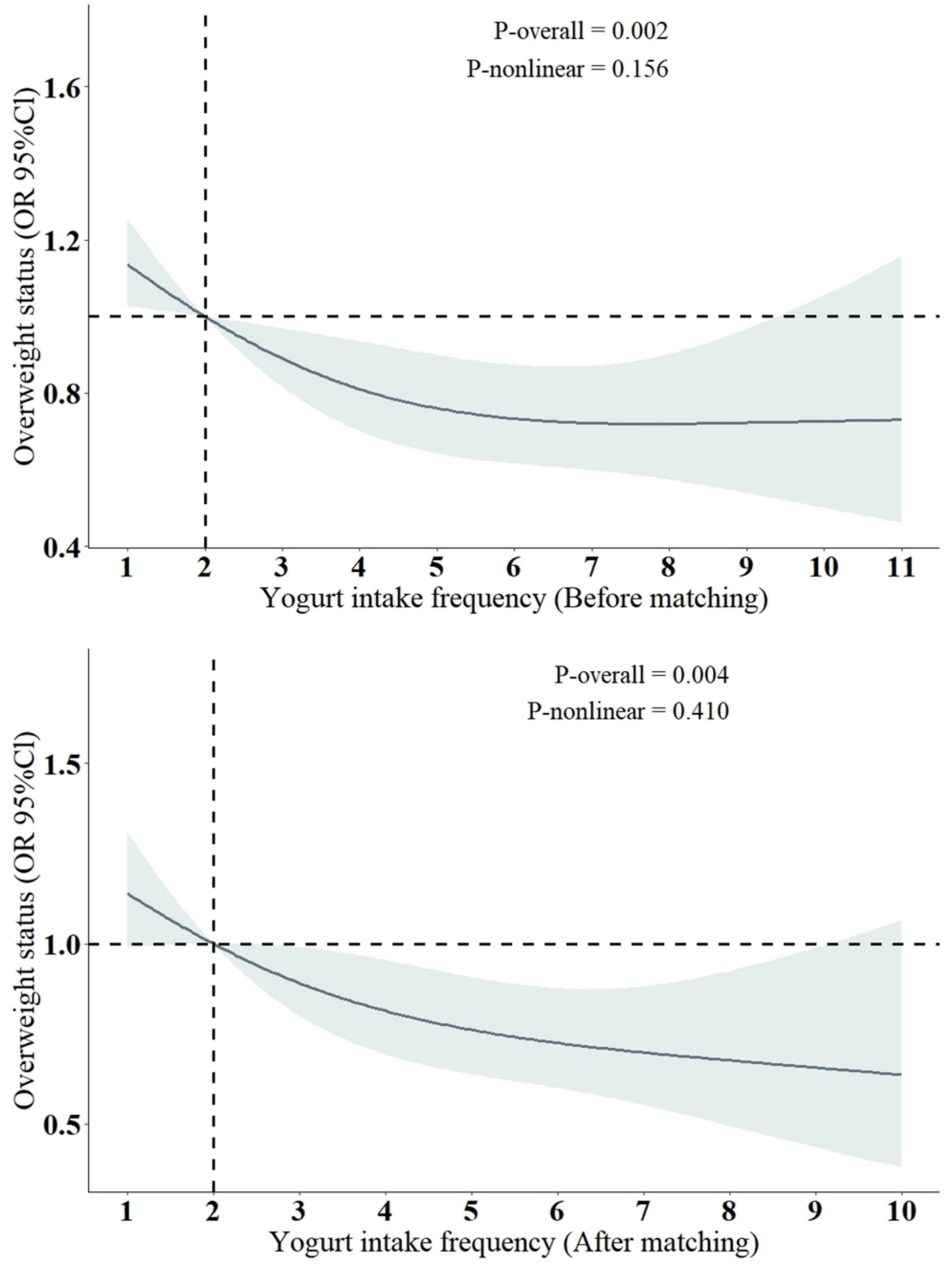
Restricted cubic spline models for the relationship between yogurt intake frequency and the risk of overweight status. OR, odds ratio; CI, confidence interval. All models were adjusted for the following covariates: sex, age, log2 cotinine, LDL, TCHOL, PIR, race/ethnicity (Mexican American, Non-Hispanic White, Non-Hispanic Black, Other Hispanic, Other race), education level (less than high school, high school or equivalent, more than high school), marital status (non-single, single), alcohol consumption (yes, no), diabetes (yes, no), high blood pressure (yes, no), coronary heart disease (yes, no), and cancer (yes, no).

Previous studies have shown a close association between yogurt consumption and BMI (or overweight status) (26–29), and there is also a strong link between BMI (or overweight status) and accelerated aging (30–33). In other words, yogurt consumption is associated with lower BMI, while higher BMI is associated with accelerated aging. Considering our findings, we have reason to infer that yogurt consumption may help delay aging, with BMI potentially playing an important mediating role in this process. Therefore, we conducted an association analysis between yogurt consumption and overweight status (defined as BMI >25). The results indicate that there is no significant association between yogurt consumption (yogurt consumer or non-consumer) and overweight status. However, there is a negative correlation between the frequency of yogurt consumption and overweight status. We also performed a nonlinear analysis of yogurt consumption frequency and overweight status. Since the PSM grouping in this study was based on whether individuals were yogurt consumers, to reduce potential bias from this grouping, we analyzed the results both before and after PSM matching, and the findings remained consistent. As shown in Table 2 and Figure 4.

**Table 2:** Association between yogurt consumption and overweight status.

Variables	OR (95% CI)	*p*-value
Overweight status by yogurt consumption status (before PSM)		
Non-yogurt consumer	Ref	
Yogurt consumer	0.732 (0.518 to 1.036)	0.106
Overweight status by yogurt consumption status (after PSM)		
Non-yogurt consumer	Ref	
Yogurt consumer	0.824 (0.629 to 1.078)	0.184
Yogurt intake frequency and overweight status (before PSM)		
Yogurt intake frequency	0.941 (0.897 to 0.988)	0.032
Yogurt intake frequency and overweight status (after PSM)		
Yogurt intake frequency	0.927 (0.871 to 0.987)	0.037

OR, odds ratio; CI, confidence interval; PSM, propensity score matching. All models were adjusted for the following covariates: sex, age, log2 cotinine, LDL, TCHOL, PIR, race/ethnicity (Mexican American, Non-Hispanic White, Non-Hispanic Black, Other Hispanic, Other race), education level (less than high school, high school or equivalent, more than high school), marital status (non-single, single), alcohol consumption (yes, no), diabetes (yes, no), high blood pressure (yes, no), coronary heart disease (yes, no), and cancer (yes, no).

The results of the RCS analysis indicate that there is primarily a negative linear association between yogurt consumption frequency and the risk of being overweight. However, since the PSM grouping in this study was based on “yogurt consumers or non-consumers” rather than the specific frequency of yogurt consumption related to overweight status, we did not further explore the potential mediating role of overweight status in the relationship between yogurt consumption and accelerated aging. This aspect requires further research for validation.

To our knowledge, this is the first study to investigate the relationship between yogurt consumption and accelerated aging. Compared to previous studies that examined yogurt consumption and its association with other diseases (24, 25), we selected a new variable—yogurt consumption frequency as the independent variable, rather than yogurt intake from the 24-h dietary recall questionnaire. This allowed us to explore the potential benefits of yogurt consumption from a new perspective. Our findings suggest that yogurt consumers have a lower risk of accelerated aging compared to non-consumers. Moreover, we observed a U-shaped curve in the association between yogurt intake frequency and the risk of accelerated aging, indicating that an appropriate frequency of yogurt intake may help delay aging.

BMI (or overweight) is one of the key risk factors for accelerated aging (30–33). Previous studies have found a close association between yogurt consumption and lower BMI, and our findings also indicate a negative correlation between yogurt consumption frequency and overweight status. Therefore, we believe that BMI may play an important mediating role in the relationship between yogurt consumption and accelerated aging.

As for the specific mechanisms underlying the association between yogurt and accelerated aging, probiotics are likely a key factor. Yogurt is rich in probiotics, which can enhance gut barrier function by increasing the number of beneficial bacteria in the gut. Additionally, probiotics can regulate immune responses and have therapeutic effects on systemic conditions such as metabolic and neurological diseases (34–36). These effects align with the health benefits of yogurt observed in other studies, suggesting that probiotics may be one of the reasons yogurt offers protective effects against various diseases. A meta-analysis on probiotics, prebiotics, and synbiotics (37) (*n* = 1,309) indicated that such microbial therapies can significantly reduce BMI in patients with non-alcoholic fatty liver disease (−0.37 kg/m^2^; 95% CI: −0.46 to −0.28; *p* < 0.001). Another prospective study on probiotics and overweight individuals also demonstrated that long-term (90 days) probiotic supplementation effectively reduces body weight. Thus, one possible link is that probiotics influence BMI by regulating the gut microbiota, and BMI, in turn, affects accelerated aging. This is the mechanism we currently consider plausible.

Given the close relationships between aging, BMI, and various diseases, we believe that yogurt intake may also have potential benefits for these diseases, presenting another promising research direction. For example, yogurt intake has been associated with kidney disease (38), sleep disturbances (25), and depression (39).

Lastly, the limitations of this study include the time constraints of the data source and the relatively small sample size. Since the NHANES database includes yogurt-related data mainly from 24-h dietary recalls and the 2003–2006 annual dietary consumption frequency, we were unable to analyze both yogurt consumption frequency and intake amount simultaneously. Further research is needed to determine the optimal yogurt consumption frequency and quantity. Additionally, because yogurt consumption frequency in this study did not specify the type of yogurt, the results may be biased due to differences in the nutritional content (e.g., sugar levels) of different types of yogurt. It is also worth noting that some studies have shown that yogurt consumers tend to eat healthier foods and have better overall lifestyle habits and dietary quality (9), which may significantly influence aging. Therefore, this study requires further validation with longer study durations and larger sample sizes.

## 4 Conclusion

Our study indicates that yogurt consumers have a lower risk of accelerated aging compared to non-consumers, with BMI likely playing a key mediating role. Therefore, an appropriate frequency of yogurt consumption may contribute to delaying the aging process. This information is valuable for the design and execution of future prospective studies aimed at delaying aging and preventing diseases through dietary interventions.

## Data Availability

The original contributions presented in the study are included in the article/supplementary material, further inquiries can be directed to the corresponding author.

## Footnotes

^1^ https://www.cdc.gov/nchs/nhanes/about_nhanes.htm
